# Supplementary material for: Non-covalent control of spin-state in metal-organic complex by positioning on N-doped graphene
Source: Nat Commun. 2018 Jul 19;9:2831. doi: 10.1038/s41467-018-05163-y (PMC6053383; doi:10.1038/s41467-018-05163-y)
Supplement: Supplementary file 1 — Supplementary Information [file 41467_2018_5163_MOESM1_ESM.pdf]

Supplementary Information:

Non-Covalent Control of Spin-State in Metal-Organic Complex by Positioning on N-Doped Graphene

Authors: Bruno de la Torre et al.

Supplementary Methods

Cluster calculations

We employ circumcircumcoronene ($C_{96}H_{24}$, abbreviation DCC) as an initial guess model for pristine graphene (Fig. S1a). Iron(II) phthalocyanine (FePc) is positioned above the surface of DCC in such a manner that there is no chance of edge-edge interaction. Furthermore, a comparatively large model ($C_{240}H_{38}$) is chosen to see the lateral movement of FePc on surface. Single graphitic N-doped model (N-DCC) for this particular study has been designed by replacing one C atom in circumcircumcoronene with one N atom (Fig. S1b), the resulting system is by one electron richer (S = ½). Additionally, pyridine-type N-doped graphene and *para* N-defect were also modeled in circumcircumcoronene molecule to make closed shell (S = 0) N-doped graphene, see Figures S1c-d. All the structures reported in this study were optimised at the DFT-D3/B97D/TZVPP [1,2] level of theory with the TURBOMOLE 6.6 suite of programs [3] and without any symmetry restrictions. Spin-unrestricted DFT was used for the open shell systems. The optimized structures are shown in Supplementary Figure S3. Grimme's advanced dispersion-corrected approach [4] (DFT-D3) with the TZVPP basis set is considered. The DFT-D3 calculation was automated using the Cuby framework [5].

In the present calculations, we have not included the correction for basis set superposition error (BSSE) in the interaction energies. The atomic charges are calculated according to the Merz-Singh-Kollman scheme [6], where the charges are fitted to reproduce the molecular electrostatic potential (MEP) at a number of points around the molecule. NBO analysis is accomplished from NBO 3.1 program [7] as implemented in Gaussian 09 [8] using the optimized geometries at B97D/def2-SVP level.

To support the experimental fact (KPFM) that there is no charge transfer between the surface and FePc, we additionally calculated the energies with different spins using constrained DFT (cDFT) developed by Van Voorhis and co-workers [9] using NWCHEM version 6.6 [10]. Remarkably, there is still spin change for FePc in presence of N-dopant. So, we can exclude the intermolecular charge transfer as a possible reason of spin crossover. All the cDFT calculations have been performed at PBE/6-31G* level [11,12].

To obtain more insight into variation of the electronic structure and triplet/singlet transition in the proximity of graphitic N-dopant we performed an analysis of molecular orbitals with the iron *d*-atomic orbitals having *z*-component (d_{z2} ,d_{xz} ,d_{yz}) of FePc on pristine (ground state having S = 1) and graphitic N-doped (ground state for FePc with S = 0) graphenes. Figure S2 displays the reorganization of the selected molecular orbitals obtained from the DFT calculations with B97D functional. We should note that this picture of d_z-component orbital reordering may be sensitive to the choice of DFT functional. This points out inherent limits of single-particle DFT methods to

describe correctly the complex interaction mechanism of FePc with the graphitic N-dopant. Unfortunately, the size of the systems prevents us to use more accurate but computationally demanding many-body methods such as CCSD(T), CASPT2, MRCI, GW, or RPA.

Periodic-boundary calculations

The spin-state and the theoretical electronic structure of FePc supported on both graphitic-N and *para* N-doped graphene were examined using the VASP (Vienna Ab initio Simulation Package) implementation of DFT in the projected augmented plane-wave scheme [13, 14]. The plane-wave cutoff energy was set to 300 eV. The *k*-space integrations were performed using the tetrahedron method [15] and a 4 × 4 × 1 Γ-centered *k*-point mesh. The computational slab included 10 × 10 graphene unit cells intercalated by a 20 Å wide vacuum layer. The positions of all atoms in the molecule and the position and distance of FePc with respect to the substrate were fully optimized using a quasi-Newton algorithm until the residual atomic forces were lower than 10 meV $Å^{-1}$ with an electronic energy convergence threshold of 10^{-6} eV. The *z*-coordinates of substrate atoms were kept fixed to prevent buckling of the graphene sheet. The optimized structures are shown in Supplementary Figure S4.
Two series of calculations were performed, (*i*) PBE-D3+*U* [12, 16] ($U_{eff} = U - J$ was chosen to be 4 eV in all cases) and vdW corrections using the scheme of Grimme *et al*. [4]; and (*ii*) non-local correlation functional optB86b+*U* [17]. The former set-up predicted the low-spin singlet state (S = 0) of FePc near graphitic N to be by about 10 kcal/mol less stable than the spin-triplet (S = 1). In contrast, the latter set-up reversed the preferred spin state in favor of the singlet state (also by ~10 kcal/mol), while predicted the molecule in the triplet state when located over the *para* N-defect fully supporting the cluster calculations and providing a plausible explanation of the experimental findings.

Multi reference calculations

Multi configurational self-consistent field (MCSCF) calculations have been carried out using Molpro 2010.1 version [18]. Since MCSCF calculations are computationally costly, here we considered only the DFT optimized free-standing FePc triplet and singlet molecules. For both the cases 8 active electrons have been considered in 10 active orbitals. Split valance polarization basis sets [19] have been used for all the atoms.

AFM simulations

The Δf images were simulated with probe particle model including the electrostatic force, based on the methods described in [20]. Pair-wise interaction were described with Morse potential, since the usage of this potential gives simulations closer to the experiment then standard Lennard Jones potentials. We used the following parameters of the flexible probe-particle tip model: the effective lateral stiffness k = 0.24 N/m and effective atomic radius R_c = 1.661 Å. We added a quadrupole-like charge distribution at the tip apex to simulate the CO-tip for all the AFM simulations [21] (quadrupole charge of -0.05×0.71^2 $e\times Å^2$). The input electrostatic potentials of the system employed in AFM simulations, was obtained from MCSCF calculations of a freestanding

molecule. Very similar distribution of total electron density and the high-resolution AFM contrast can be also obtained using the total electron densities using the cluster DFT-D3/B97D calculations. Parameters of Morse pairwise potentials for all elements are listed in Supplementary Table S2. The atomic radii for different elements/atoms were obtained from the best match to the total density of free standing FePc molecule in singlet and triplet state, respectively. The Morse potential width parameter α was set to 1.6 Å^{-1} for all pairwise interactions. (The atomic energies and C_1 radius was obtained from OPLS force-field [22]).

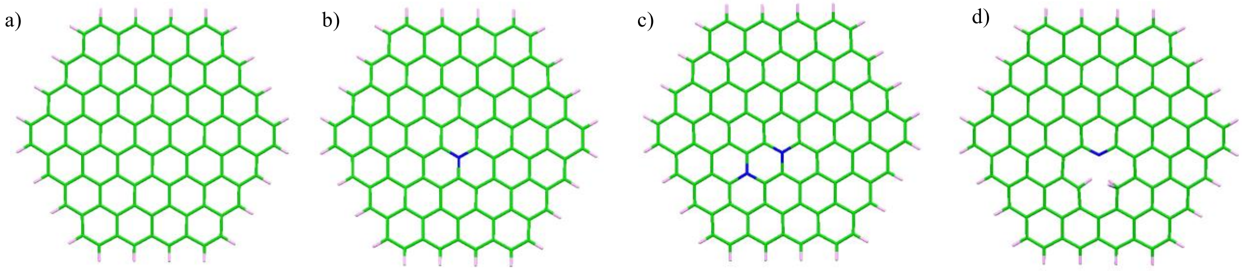

Supplementary Figure 1. Optimized geometries. (a) Circumcircumcoronene, (b) Graphitic N-doped Circumcircumcoronene, (c) Graphitic *para* N-defect Circumcircumcoronene and (d) pyridine-type N defect in circumcircumcoronene. [C: green, N: blue, H: light pink]

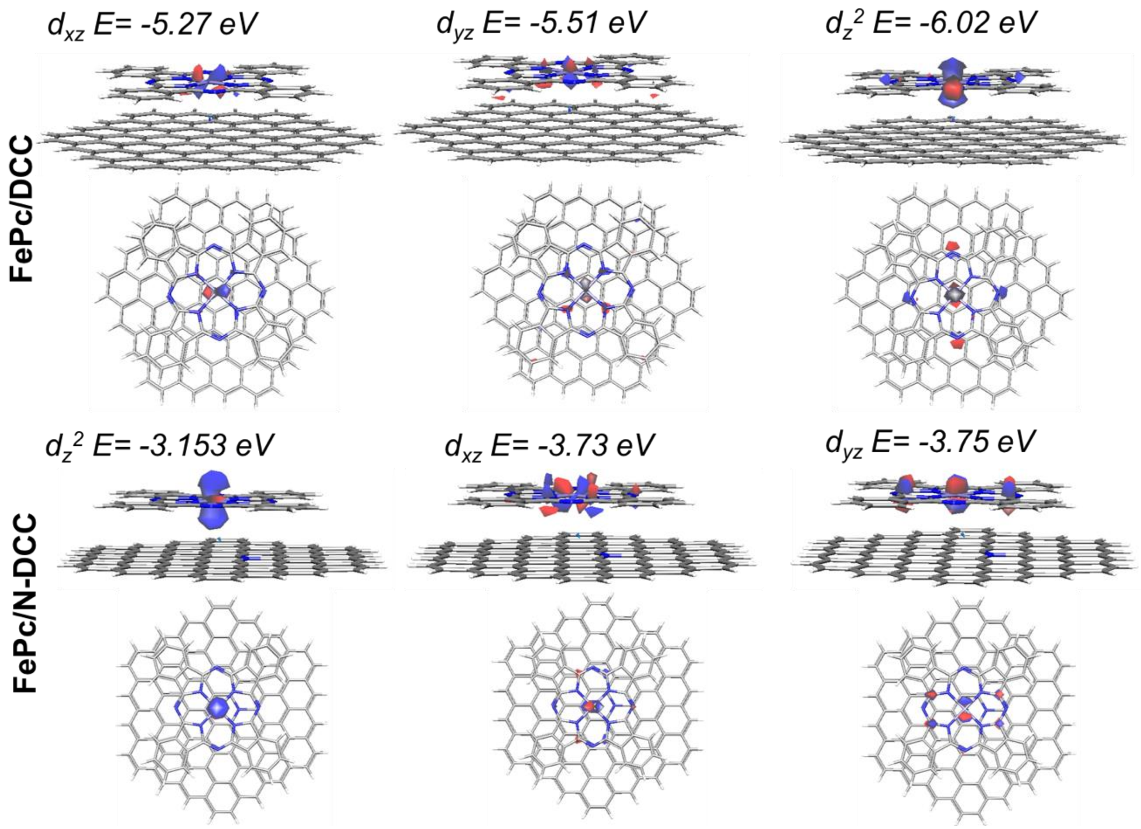

Supplementary Figure 2. Variation of selected molecular orbitals with *d_z*-component. (*d_{z2}, d_{xz}, and d_{yz}*) of FePc on pristine (Ground state, S = 1) and graphitic N-doped (Ground state, S = 0) graphene obtained from the DFT calculations with B97D functional using 0.05 iso-surface value. In FePc/DCC, molecular orbitals with *d_z*-component of FePc have contributions in occupied levels, whereas in FePc/N-DCC, molecular orbital with *d_{z2}* has mainly contributed to LUMO level and remaining two *d_z*-components (*d_{xz}, and d_{yz}*) mainly contributed to occupied levels.

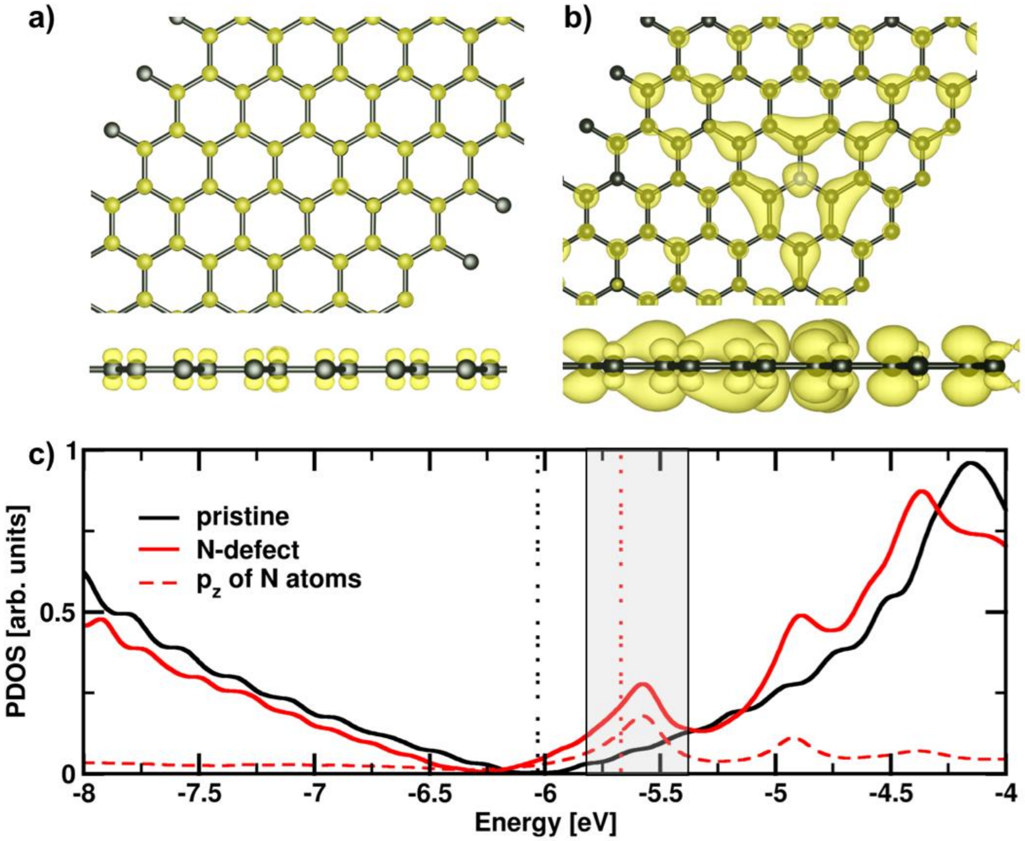

Supplementary Figure 3. Isosurfaces of pristine and N-doped graphene. Isosurfaces (0.003e) of calculated real space charge density of pristine graphene (a) and graphitic N-doped graphene (b) in the energy range from -5.8 to -5.4 eV (marked by grey transparent column in Fig. (c)); (c) calculated density of state (DOS) for the pristine and graphitic N defect. The dotted vertical lines denote the Fermi level for each system. The dashed line represents projected DOS corresponding to p_z-orbital of N atom. The results were obtained with 6x6 unit cell sampled with 144 k-points.

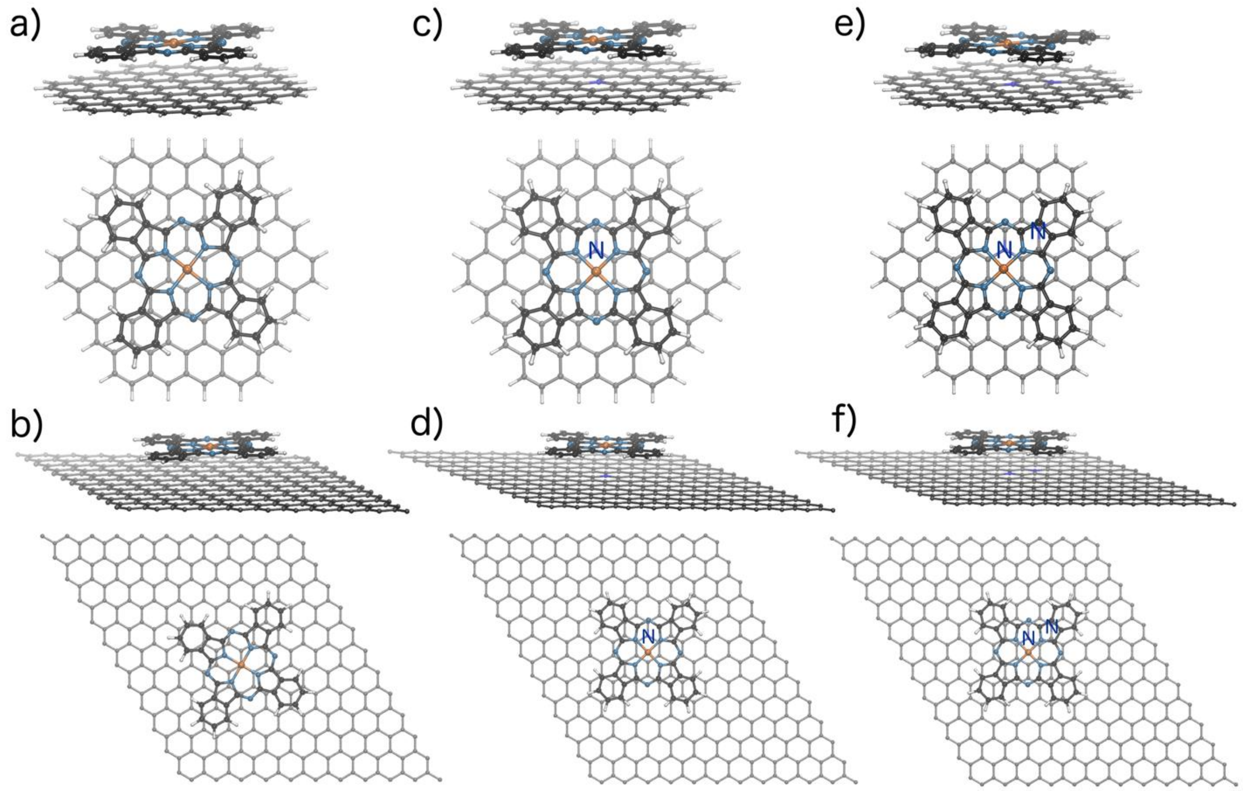

Supplementary Figure 4. Optimized atomic structures of FePc. FePc on pristine graphene (a,b), near the graphitic N and (c,d), and (e,f) over the *para* N defect obtained from the cluster and periodic calculations. C atoms are shown in grey and black, N atoms in blue, Fe in orange, and H in light-grey. Different hues are used to discriminate between the substrate's atoms and the molecule's atoms.

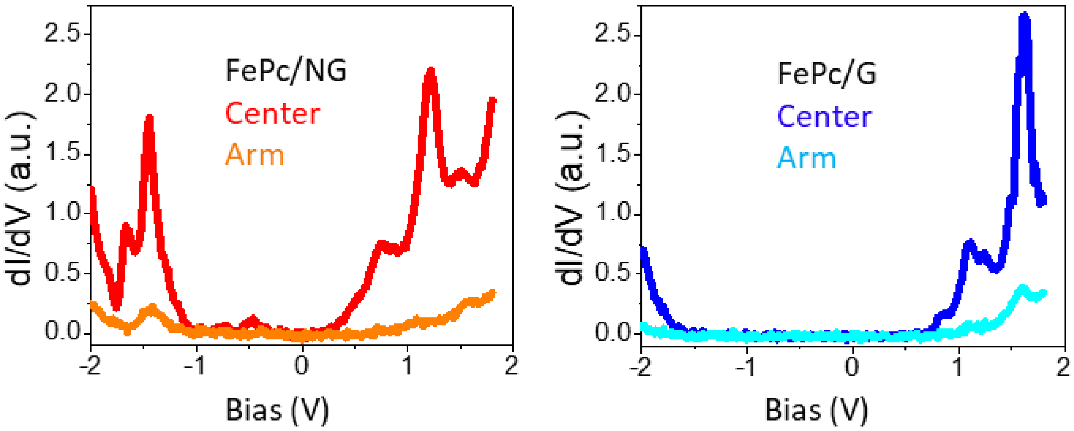

Supplementary Figure 5. d*I*/d*V* spectroscopy at the center of the FePc and a benzene ring. a) d*I*/d*V* spectroscopy acquired on FePc adsorbed at the proximity of a N-dopant, at the center (red) and on one peripheral benzene ring (orange) of the molecule. b) d*I*/d*V* spectroscopy acquired on FePc adsorbed on pristine graphene, at the center (blue) and on one peripheral benzene ring (cyan) of the molecule.

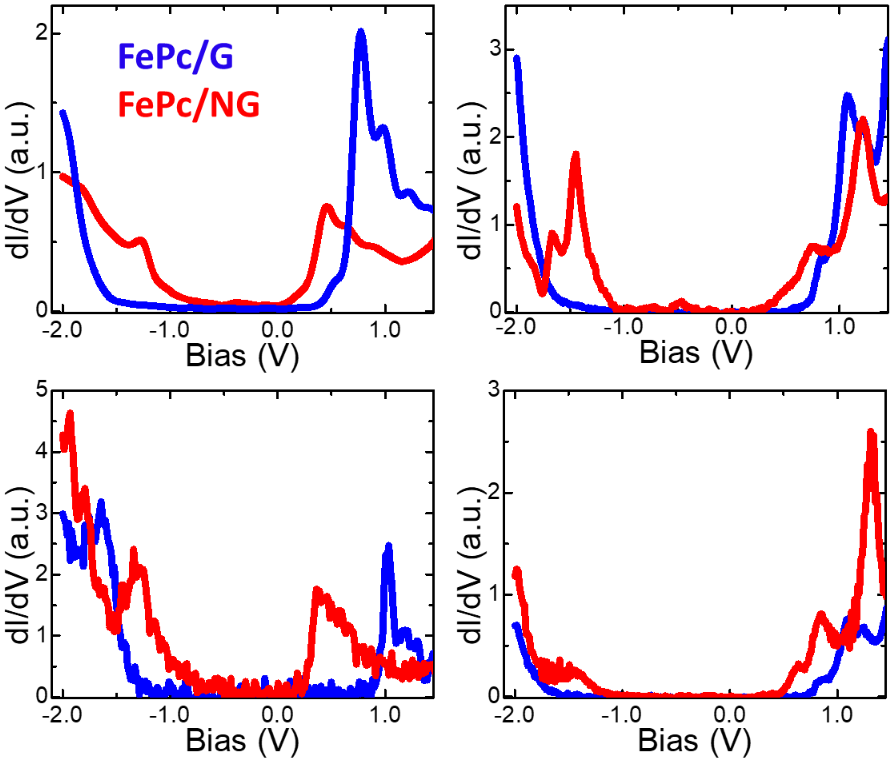

Supplementary Figure 6. Lowering the molecular gap due to the N-dopant Set of constant height d*I*/d*V* plots acquired at the center of FePc molecules adsorbed on N-dopant (red) and on pristine graphene (blue).

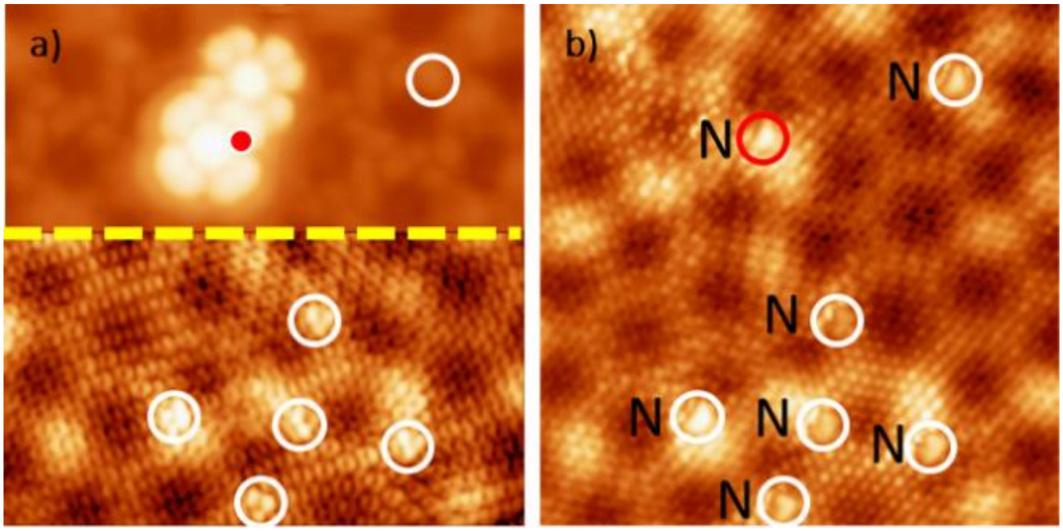

Supplementary Figure 7. Revealing the N-dopant under a FePc molecule. a) STM topography image of a FePc dimer on N-doped graphene. The top half was acquired at Vb=-2.0V and the bottom half at Vb=-0.05V. b) STM topography high-resolution image on the same region as (a) acquired after lateral manipulation of FePc molecules. Vb = -0.05V, It = 10pA.

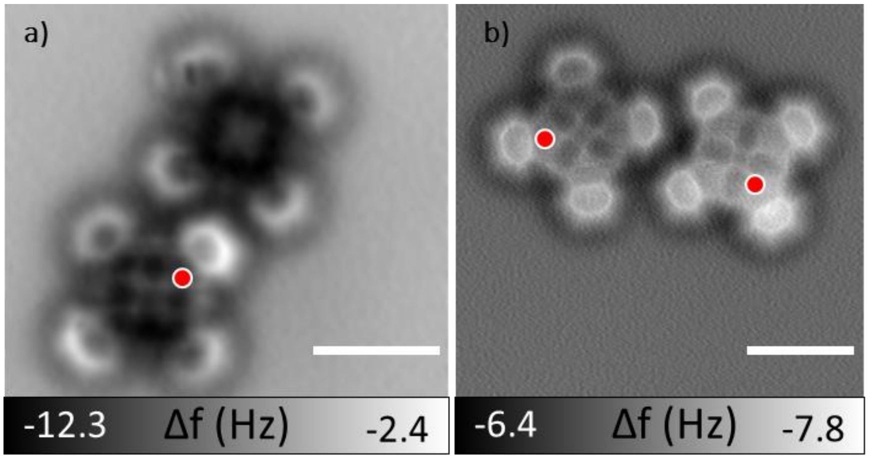

Supplementary Figure 8. High-resolution AFM images on molecules with CO-tip. a) AFM high-resolution image of a FePc dimer. The FePc molecule adsorbed near the N-dopant shows a square-like feature at the center while the molecule on pristine graphene shows a cross-like feature at the center. b) AFM high-resolution image of a FePc dimer. Both molecules are adsorbed near a N-Dopant and both show square-like features at the center. In both images, the scalebar corresponds to 1nm.

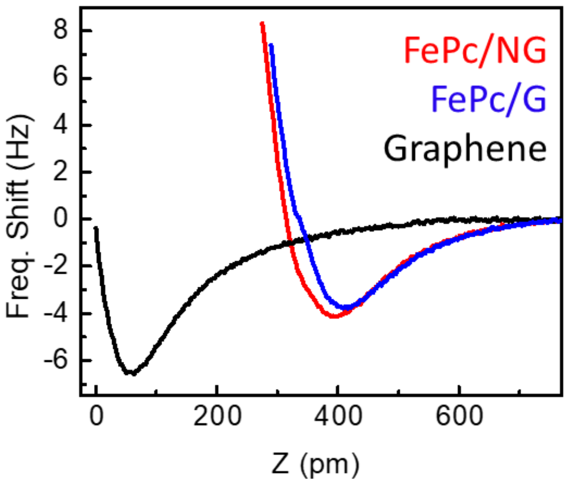

Supplementary Figure 9. Measurement of the molecular adsorption height. Freq. Shift vs. Z measured at the center of, two FePc molecules adsorbed on N-dopant and on pristine graphene, and on a carbon atom in graphene. The difference in the Z position of the frequency shift minima gives the FePc adsorption height about 335pm above the graphene substrate, regardless of the doping

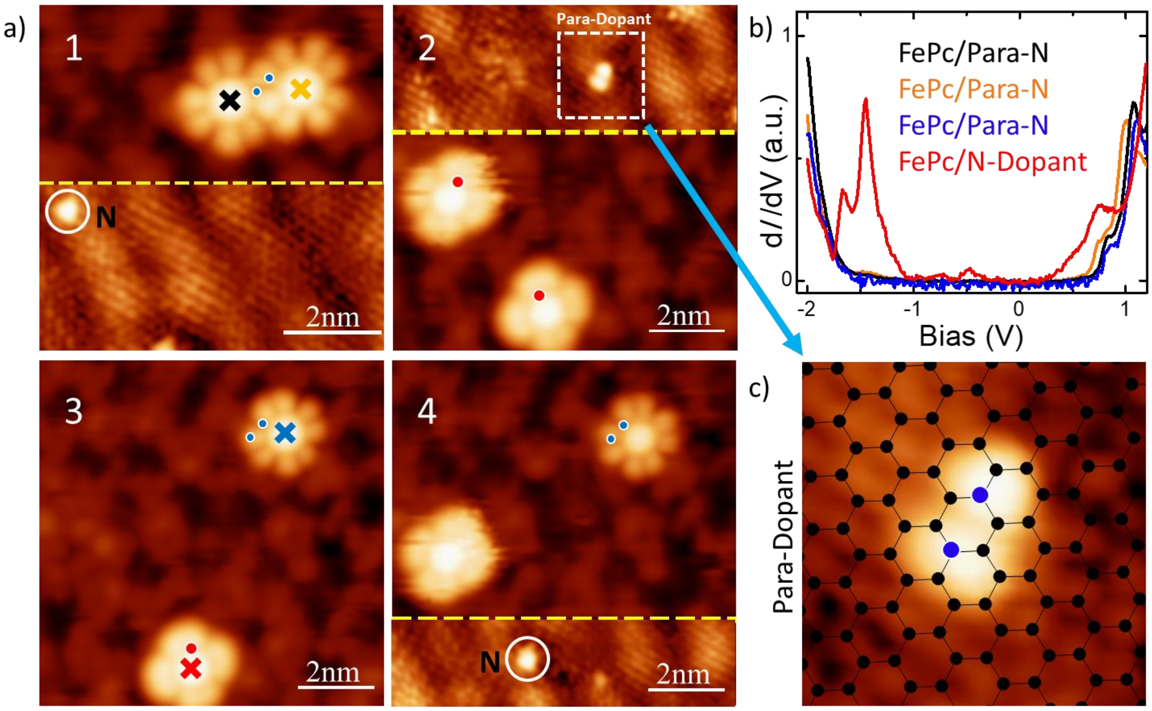

Supplementary Figure 10. Effect of substitutional para-N-dopants on FePc molecules. a) Sequence of consecutive STM images of 2 FePc molecules acquired after each manipulation step. Initially, two FePc molecules are adsorbed on a substitutional para-N-dopant. In 2, both molecules are manipulated with the STM tip and transferred to two separated single N-dopants and the exact position of the para-N-dopant is resolved. In 3 one FePc is transferred to the para-N-dopant again. Finally, in 4, one FePc molecule is displaced from one single N-dopant to another. Please note that the image in the situation 1 is smaller than others in situation 2, 3 and 4. b) dI/dV spectra acquired in situations 1 and 3 on the center of FePc molecules. On single N-dopant, FePc shows lower energy features than on the para-N-dopant, where the molecule behaves as on bare graphene. c) STM image of para-N-dopant in detail. Images were acquired with It = 10pA and Vb = -2.0V, and Vb = -0.05V when obtaining atomic resolution. The yellow dashed line indicates where the bias voltage is changed.

Cluster System	E_{int} [kcal/mol]	E_{int} from cDFT [kcal/mol]	Slab System	E_{int} [kcal/mol]
Circumcircumcoronene…FePc	-59.3	-48.1	Graphene…FePc	-62.1
Graphitic N-doped Circumcircumcoronene…FePc	-66.4	-49.8	Graphitic N-doped Graphene…FePc	-64.7
Graphitic *para* N-defect Circumcircumcoronene…FePc	-64.0	-	Graphitic *para* N-defect Graphene…FePc	-62.6
Pyridine-type N defect Circumcircumcoronene…FePc	-60.8	-	-	-

Supplementary Table 1. Interacting energies. The interaction energies for the complexes were calculated using the following equation $E_{int} = E_{ALL} – E_{FePc} – E_{surf}$, where E_{ALL} means total energy of the complex and E_{FePc} and E_{surf} are total energies of FePc molecule and graphene surface, respectively. The interaction energies E_{int} in kcal/mol were obtained from cluster DFT-D3/B97D (second column), cDFT (third column) and periodic DFT-optB86b+*U* calculations (fifth column) of various FePc complexes.

Element	ε [meV]	r [Å]
H	0.681	1.200
C1 (out)	9.106	1.908
C2 (middle)	9.106	1.945
C3 (in)	9.106	1.897
N1 (inner)	11.491	2.014
N2 (out)	11.491	1.965
Fe (singlet)	10.0	1.800
Fe (triplet)	10.0	2.000

Supplementary Table 2. Parameters of Morse pairwise potentials for AFM simulations.

References

[1] F. Weigend, M. Häser, H. Patzelt and R. Ahlrichs; Chem. Phys. Letters 294, 143 (1998)

[2] S. Grimme, J. Comp. Chem., 27, 1787 (2006).

[3] TURBOMOLE V6.6 2014, a development of University of Karlsruhe and Forschungszentrum Karlsruhe GmbH, 1989-2007, TURBOMOLE GmbH, since 2007; available from http://www.turbomole.com.

[4] S. Grimme, J. Antony, S. Ehrlich, H. Krieg, J. Chem. Phys., 132, 154104 (2010).

[5] J. Rezac, J. Comput. Chem., 2016, 37, 1230 (2016).

[6] U. C. Singh and P. A. Kollman, J. Comp. Chem., 5, 129 (1984).

[7] NBO Version 3.1, E. D.Glendening, A. E.Reed, Eds.

[8] Gaussian 09, Revision D.01, M. J. Frisch, G. W. Trucks, H. B. Schlegel, G. E. Scuseria, M. A. Robb, J. R. Cheeseman, G. Scalmani, V. Barone, B. Mennucci, G. A. Petersson, et al. Gaussian, Inc., Wallingford CT, 2009.

[9] B. Kaduk, T. Kowalczyk, and T. Van Voorhis Chem. Rev. 112, 321 (2012) and references therein.

[10] M. Valiev, E.J. Bylaska, N. Govind, K. Kowalski, T.P. Straatsma, H.J.J. van Dam, D. Wang, J. Nieplocha, E. Apra, T.L. Windus and W.A. de Jong, Comput. Phys. Commun. 181, 1477 (2010).

[11] G. A. Petersson, A. Bennett, T. G. Tensfeldt, M. A. Al-Laham, W. A. Shirley, and J. Mantzaris, J. Chem. Phys., 89 2193 (1988).

[12] P. Perdew, K. Burke, and M. Ernzerhof, Phys. Rev. Lett. 77, 3865 (1996).

[13] G. Kresse and J. Furthmüller, Comput. Mater. Sci. 6, 15 (1996).

[14] G. Kresse and D. Joubert, Phys. Rev. B 59, 1758 (1999).

[15] P. E. Blöchl, O. Jepsen, and O. K. Andersen, Phys. Rev. B 49, 16223 (1994).

[16] S. L. Dudarev, G. A. Botton, S. Y. Savrasov, C. J. Humphreys, and A. P. Sutton, Phys. Rev. B 57, 1505 (1998).

[17] J. Klimeš, D. R. Bowler, and A. Michaelides, Phys. Rev. B 83, 195131 (2011).

[18] MOLPRO, version 2010.1, a package of *ab initio* programs, H.-J. Werner, P. J. Knowles, G. Knizia, F. R. Manby, M. Schütz, and others , see http://www.molpro.net.

[19] A. Schäfer, H. Horn and R. Ahlrichs; J. Chem. Phys. 97, 2571 (1992).

[20] P. Hapala et al. Phys. Rev. B 90, 085421 (2014); P. Hapala et al. Phys. Rev. Lett. 113, 226101 (2014).

[21] J. Peng et al Nature Commun. 9, 122 (2018).

[22] W.L. Jorgensen, D.S, Maxwell, J. Tirado-Rives, JACS 118, 11225 (1996).

[23] A.J. Heinrich, J.A. Gupta, C.P. Lutz, D.M. Eigler, Single-Atom Spin-Flip Spectroscopy. *Science* 306, 466 (2004).

[24] J. Li et al., Sci. Adv. (2018); 4: eaaq0582
